# The osteogenic response of undifferentiated human mesenchymal stem cells (hMSCs) to mechanical strain is inversely related to body mass index of the donor

**DOI:** 10.3109/17453670903171883

**Published:** 2009-08-01

**Authors:** Gerald Friedl, Reinhard Windhager, Helena Schmidt, Reingard Aigner

**Affiliations:** ^1^Department of Orthopaedics and Orthopaedic Surgery, Medical University of GrazGrazAustria; ^2^Institute of Molecular Biology and Biochemistry, Center of Molecular MedicineGrazAustria; ^3^Department of Radiology, Division of Nuclear Medicine, Medical University of GrazGrazAustria

## Abstract

**Background** While the importance of physical factors in the maintenance and regeneration of bone tissue has been recognized for many years and the mechano-sensitivity of bone cells is well established, there is increasing evidence that body fat constitutes an independent risk factor for complications in bone fracture healing and aseptic loosening of implants. Although mechanical causes have been widely suggested, we hypothesized that the osteogenic mechano-response of human mesenchymal stem cells (hMSCs) may be altered in obese patients.

**Methods** We determined the phenotypic and genotypic response of undifferentiated hMSCs of 10 donors to cyclic tensile strain (CTS) under controlled in vitro conditions and analyzed the potential relationship relevant to the donor's anthropomorphometric and biochemical parameters related to donor's fat and bone metabolism.

**Results and interpretation** The osteogenic marker genes were all statistically significantly upregulated by CTS, which was accompanied by a significant increase in cell-based ALP activity. Linear correlation analysis revealed that there was a significant correlation between phenotypic CTS response and the body mass index of the donor (r = –0.91, p < 0.001) and phenotypic CTS response was also significantly related to leptin levels (r = –0.68) and estradiol levels (r = 0.67) within the bone marrow microenvironment of the donor. Such an upstream imprinting process mediated by factors tightly related to the donor's fat metabolism, which hampers the mechanosensitivity of hMSCs in obese patients, may be of pathogenetic relevance for the complications associated with obesity that are seen in orthopedic surgery.

## Introduction

With its increasing prevalence in the western world, obesity is causing an increase in the socioeconomic burden due to its harmful consequences, including its effects on the musculoskeletal system (Anandacoomarasamy et al. 2007). Obesity appears to be an independent risk factor for increased fracture risk (Strotmeyer et al. 2005, Leslie et al. 2007) and complications in fracture healing (nonunions) (Green et al. 2005, Collman et al. 2006, Hofmann et al. 2008), as well as for radiological and/or clinical implant failure following total joint replacement (Ranawat and Boachie-Adjei 1988, Stern and Insall 1990, Smith et al. 1992, Griffin et al. 1998, Winiarsky et al. 1998, Vazquez-Vela et al. 2003, Foran et al. 2004, Berend et al. 2005, Amin et al. 2006, Gillespie and Porteous 2007). While mechanical reasons (due to “overload”) have been widely suggested, our knowledge about the pathogenesis at the cellular level is still very limited.

The ability of hMSCs to differentiate into several mesenchymal cell lineages including the osteoblast lineage plays a key role in skeletogenesis and bone regeneration throughout life, and biological factors such as cell recruitment, proliferation, and differentiation have been considered to be critical in this regard. However, the differentiation of hMSCs is a highly programmed lineage-specific process (Kulterer et al. 2007) triggered by microenvironmental factors including hormones, cytokines, and growth factors (Tuan et al. 2003)—but importantly, also by biomechanical conditions. Indeed, it has been shown recently that tensile forces will not only support but rather inherently induce the osteogenic differentiation of undifferentiated hMSCs under appropriate in vitro conditions (Friedl et al. 2007, Mirza et al. 2007).

Several authors have considered the importance of the host microenvironment in the differentiation process of hMSCs (Kuznetsov et al. 1997, Caplan et al. 1998), although the clinical relevance of donor-related variability still remains elusive. For example, some studies have shown that the functional characteristics of hMSCs may be profoundly affected under clinical conditions of osteoporosis (Rodriguez et al. 1999, Mendes et al. 2002), as was also reported for alcohol-induced osteonecrosis (Suh et al. 2005) and osteoarthritis (Lisignoli et al. 2004), thus suggesting that hMSCs may have a critical role in the pathogenesis of these diseases. With regard to the obesity-associated incidence previously mentioned, we hypothesized that the initial osteogenic mechano-response of undifferentiated hMSCs may be profoundly affected by physiological conditions related to the donor's fat metabolism (the null hypothesis being that there are no differences in the osteogenic response of hMSCs between obese and non-obese donors).

## Material and methods

### Experimental design

Bone marrow-derived hMSCs were isolated from 5 female and 5 male age-matched donors undergoing elective orthopedic surgery. To obtain cells in an undifferentiated state, hMSCs were expanded under standard culture conditions for cell growth, which was recently demonstrated to keep the cells in an undifferentiated state up to passage 10 (Kulterer et al. 2007). In addition, the cells were seeded at low (subconfluent) cell density to minimize contact inhibition and spontaneous differentiation. In an effort to eliminate possible confounding factors that might affect cell differentiation unrelated to mechanical load, the individual response of undifferentiated hMSCs to cyclic tensile strain (CTS) was determined using a two-armed study design (strained vs. unstrained under otherwise equal in vitro conditions). Mechanical stimulation was applied with a computer-assisted 4-point bending device, for which uniaxial elongation has been shown to be transferred properly to cell monolayers (Neidlinger-Wilke et al. 2001). The magnitude of tensile strain was restricted to a maximum of 3,000 microstrain (μE) in order to avoid a pathological response that has been found previously in monolayer culture systems due to the application of supra-physiological strains (Murray and Rushton 1990).

Phenotypic effects were characterized by analyzing cell numbers, cell viability, and ALP activity. The mRNA levels of marker genes for early osteogenic differentiation (RUNX2, ALPL, SPARC, SPP1), protein synthesis (COL1A1), and cell cycle (MKI67) were determined by real-time RT-PCR. To investigate donor-related variability affecting the osteogenic potential of hMSCs, linear correlation analysis was performed between the functional characteristics and donor-specific physiological characteristics, including various biochemical indices for donor's bone and fat metabolism.

### Subjects

The study protocol was approved by the local Institutional Review Board (control no. 12-091) and informed consent was obtained from all subjects. Exclusion criteria included a history of myelogenous disease, a current history of neoplasm, or infections. All patients had normal renal function (serum urea, serum creatinine, and creatinine clearance) and did not suffer from any conditions known to seriously affect bone metabolism (rheumatoid arthritis, morbus Paget, diabetes mellitus) or associated with severe osteoporosis (i.e. ongoing steroid intake, anti-resorptive or bone anabolic treatment).

### MSC isolation and cyclic tensile strain (CTS)

Cell isolation and expansion was performed as previously described (Friedl et al. 2007). Briefly, less than 10 mL heparinized bone marrow aspirate was harvested from the iliac crest at the start of elective surgery. Mononuclear cells were isolated by gradient centrifugation and expanded under standard conditions in complete growth medium: DMEM/F12 with 10% fetal bovine serum (FBS) (lot no. 40F6120K; Gibco, Vienna, Austria), 2 mM L-glutamine, 100 U/mL penicillin and streptomycin, and 1 ng/mL amphotericin B (Sigma-Aldrich, Vienna, Austria) until passage 4–6. FACS analysis showed the following surface protein expression pattern to be characteristic of hMSCs used in the experiment (Kulterer et al. 2007): CD14^–^, CD29^+^, CD34^–^, CD44^+^, CD45^–^, CD49e^+^, CD105^+^, CD166^+^, without any significant quantitative differences between donors (Serotec, Vienna, Austria).

Custom-made silastic dishes were preconditioned with standard growth medium, washed twice with PBS, and coated with fibronectin (5 μg/mL in PBS; PAA, Pasching, Austria) for 20 min at room temperature. hMSCs were plated at a cell density of 1 × 10^4^ cells per cm^2^ after careful washing. The cells were allowed to attach overnight in growth medium supplemented with 0.05 mM L-ascorbic acid 2-phosphate (Sigma-Aldrich, Vienna, Austria) before starting CTS. The same procedure without the application of cyclic tensile strain was performed simultaneously in controls (CON).

CTS was applied with a computerized custom-made 4-point bending device (Bottlang et al. 1997) (Cell Strain Unit version 3.0, University of Iowa, IA), as tensile strains of less than 4,000 μstrain have been shown to be related to the level associated with the physiological process of bone (re)modeling (Murray and Rushton 1990). The following straining protocol was used: 1,000 cycles (1 Hz) of sawtooth-shaped uniaxial tensile strain with a maximum of 3,000 μstrain, 6 times a day for a total period of 72 h. Culture medium was changed on day 2 and any appearance of non-adherent cells was checked by careful examination of the supernatant by light microscopy. All measurements started immediately after the last straining cycle and they were performed at least in triplicate.

### Cell proliferation and cell viability

Cell viability was tested with the XTT assay (Cell Proliferation Kit II; Roche Diagnostics, Mannheim, Germany) without detaching the cells, following the manufacturer's instructions. In addition, resuspended cells were stained with trypan blue, and at least 100 viable cells were counted in duplicate by two independent investigators using a hemocytometer. No significant differences were found in cell viability between strained and unstrained hMSCs (86% (SD 10) and 89%, (SD 8.5), respectively).

### Analysis of mRNA expression by real-time RT-PCR

After completion of the last training cycle, total RNA isolation was started immediately using the Aqua Pure RNA Isolation Kit (Bio-Rad, Vienna, Austria) according to the manufacturer's instructions. RT-PCR was performed as previously described (Friedl et al. 2007). Briefly, reverse transcription was carried out after treatment with DNAse I (Sigma-Aldrich, Vienna, Austria) using the 1st Strand cDNA Synthesis Kit (Roche Diagnostics, Mannheim, Germany) with random hexamer primers reverse transcription. Negative controls were set up without RNA templates and without reverse transcriptase (AMV). Preparations of cDNA were checked by spectrophotometry (at A_260_ and A_280_) and by gel electrophoresis. The PCR primers (TIB-Molbiol, Berlin, Austria) used, the product length, and estimated melting temperatures are listed in Table 1. Real-time PCR was run in a LightCycler Instrument (Roche, Germany) using the FastStart DNA Master SYBR Green I Kit (Roche Diagnostics, Mannheim, Germany) with an initial activation of the FastStart Taq polymerase at 95°C for 10 min. Amplification conditions were 35 cycles of annealing at 56–58°C for 7 seconds followed by an extension phase at 72°C for 7–9 seconds. The PCR products were visualized by agarose gel electrophoresis and sequenced by fluorescent automated sequencing (Beckman model CEQ8000, software version 5-0.345). The relative changes in gene expression were determined by the 2^-ΔΔCT^ method as described in detail by Livak and Schmittgen (2001). Normalization was performed to the mRNA level of clathrin (CLCT) and B2M, which produced essentially the same results.

**Table 1.** Primers used for real-time RT-PCR

Symbol	Gene name	Accession no.	Primer sequence	Amplicon size (bp)	MT (°C)
CLTC	clathrin, heavy polypeptide (Hc)	X55878	5'-AGAAACTGCATGGAGGCACAA	165	81
			3'-TGGGGCTGACCATAAACAATG		
B2M	beta-2-microglobulin	AB021288	5'-GCTATCCAGCGTACTCCAAAGA	102	80
			3'-GGATGGATGAAACCCAGACA		
COL1A1	collagen, type I, alpha 1	Z74615	5'-CGAAGACATCCCACCAATCAC	250	92
			3'-TCCCTTGGGTCCCTCGAC		
MKI67	antigen identified by monoclonal antibody Ki-67	X65550	5'-GCCTGTACGGCTAAAACATGGA	182	82
			3'-TTGAGGAGAGGCAGGGTGAA		
RUNX2	runt-related transcription factor 2	AF001443	5'-ACAGTAGATGGACCTCGGGAAC	82	83
			3'-TGAGGCGGTCAGAGAACAAA		
ALPL	alkaline phosphatase, liver/bone/kidney	NM_000478	5'-CCTGGACCTCGTTGACACCT	136	86
			3'-GTCCCCTGGCTCGAAGAGA		
SPARC	secreted protein, acidic, cysteine-rich (osteonectin)	J03040	5'-TGCCACTGAGGGTTCCCA	211	88
			3'-TCGGTTTCCTCTGCACCATC		
SPP1	secreted phosphoprotein 1 (osteopontin, bone sialoprotein I, early T-lymphocyte activation 1)	NM_000582	5'-AACGCCGACCAAGGAAAACT	150	84
			3'-GGCCACAGCATCTGGGTATT		
COL2A1	Collagen, type II, alpha1	MN_001844	5'-ATGCTGGTCCTCAAGGCAAA	196	93
			3'-CCAGGAAGACCCCTCAGACC		
CSPCP	Homo sapiens aggrecan	MN_001135	5'-CGAGGAGCAGGAGTTTGTCA116	87	
			3'-TCTCAAATTGCATGGGGTGT		

### Alkaline phosphatase (ALP) activity

ALP activity was measured with standard protocols for conversion of p-nitrophenyl phosphate (p-NPP) to p-nitrophenol (p-NP) (Sigma Kit 104, Vienna, Austria). Cells were lysed with 0.5% Triton X-100 (Sigma-Aldrich, Vienna, Austria) after triple washing with Tyrode's balanced salt solution, and lysates were incubated with 360 μM p-NPP in 0.75 M ALP buffer (pH 10.3) at 37°C. Quantification of p-NP was carried out kinetically within a linear range for 10 min at 405 nm using a p-NP standard absorption curve. Diluted FBS was used as positive control. Measurements were performed in triplicate for each of three 2.8-cm^2^ wells, and the mean values were used for calculations. Results are expressed either as μmol p-NP/min per well (μM/min) or as cell-based values by normalizing to cell numbers (μM/min/10^6^ cells).

### Bone marrow plasma measurements

Biochemical measurements were performed on bone marrow plasma because it constitutes the humoral microenvironment of hMSCs in vivo. Fasting plasma was isolated by centrifugation of heparinized bone marrow aspirates and it was stored at –80°C until use.

All measurements including triglycerides, cholesterol, glucose 17β-estradiol (Adaltis, Bologna, Italy), leptin (Diagnostic System Laboratories, Sinsheim, Germany), resistin and adiponectin (R&D Systems, distributed by Biomedica Gruppe, Vienna, Austria), CrossLaps (Nordic Bioscience Diagnostic, Herlev, Denmark), osteocalcin (CIS bio international, Gif-sur-Yvette Cedex, France), osteoprotegerin (Immunodiagnostic Systems Ltd., Boldon, UK), insulin-like growth factor I (IGF-I), intact parathyroid hormone [1-84], and human growth hormone (all R&D Systems, Biomedica Gruppe, Vienna, Austria) were performed by the central laboratory core unit of the university hospital in duplicate; mean values were used for analysis.

### Data analysis

Deviations from normal distribution were tested with the Kolmogorov-Smirnov test. Statistical analyses were performed with the paired-samples t-test, differential gene expression was analyzed with the Wilcoxon signed-ranks test, and correlations were tested by Pearson r statistics. Possible differences between female and male donors were analyzed by two-way ANOVA analysis, and values are presented as either mean (with SEM or SD) or median value with corresponding confidence interval as appropriate. All statistics were performed with SigmaStat version 2.03.0 (SPSS Inc.) and results were considered significant at p < 0.05.

## Results

### Donor and baseline characteristics

5 female donors (f, aged 65 (SD 13) years) and 5 male donors (m, aged 67 (SD 17) years) with a mean body mass index (BMI) of 30 (SD 6.0) kg/m^2^ were included (Table 2). In our cohort, donor's age was significantly associated with leptin levels (r = 0.67, p < 0.05) and tended to be inversely related to human growth hormone levels (r = –0.62, p = 0.06). While there was a weak trend of an association between donor's age and BMI (r = 0.51, p = 0.1), there was a significant correlation between the latter and plasma leptin levels (r = 0.81, p = 0.0001) and an inverse correlation with IGF-I levels (r = –0.77, p = 0.007). No significant correlations were found between the anthropomorphometric characteristics and any of the other indices listed in Table 4.

**Table 2.** Characteristics of donors 5 female and 5 male donors matched for age were included consecutively and bone marrow-derived human mesenchymal stem cells (hMSCs) from passage no. (p) 4–6 were used for in vitro experiments

Donor	Sex	Age	BMI	Diagnosis	Surgery	hMSCs
01	f	53	18.7	Osteoarthritis hip	Total hip arthroplasty	p5
02	f	77	25.6	Osteoarthritis hip	Total hip arthroplasty	p4
03	f	64	35.3	TJR hip – aseptic loosening	Revision of cup	p4
04	m	73	24.2	Osteoarthritis knee	Total hip arthroplasty	p6
05	m	81	30.0	Osteoarthritis hip	Total hip arthroplasty	p4
06	m	39	23.3	Idiopathic scoliosis	Scoliosis correction surgery	p4
07	m	75	37.6	TJR hip – aseptic loosening	Revision of stem	p4
08	f	78	32.9	TJR knee – aseptic loosening	Revision, exchange of tibial component	p4
09	m	68	25.8	Osteoarthritis hip	Total hip arthroplasty	p4
10	f	52	25.9	Osteoarthritis hip	Total hip arthroplasty	p4

### Biochemical and molecular characteristics of unstrained hMSCs (CON)

Cell-based ALP activity ranged between 0.89 and 17 μM/min/10^6^ cells (mean 6.2 (SD 5.0) μM/min/10^6^) in control hMSCs (CON), while donor-to-donor variability in marker gene expression showed differences of up to 48 fold in the message levels of mRNA. There were no significant correlations between the genotypic or phenotypic characteristics of control hMSCs and any of the donor-specific parameters listed in Table 4 (including gender; data not shown).

### Effects of cyclic tensile strain (CTS)

**Figure 1.**
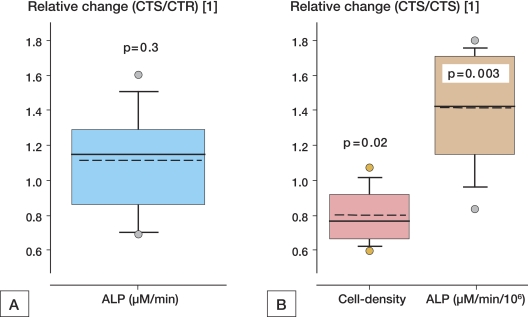
Osteogenic response of hMSCs to mechanical strain. A. The phenotypic response expressed as relative change in ALP activity between unstrained (CTR) and strained (CTS) hMSCs at a culture-well based level (μM/min) accounts for both. B. The effects on cell-density and cell-based ALP activity. Dashed lines show mean values.

The early osteogenic marker genes were all significantly upregulated due to CTS: i.e. RUNX2 (1.9-fold), ALPL (2.7-fold), SPARC (4.2-fold), SPP1 (2.8-fold). COL1A1 expression was also significantly enhanced (3.5-fold), while the median value of the messages of cell cycle-related antigen Ki67 tended to be decreased (Table 3). This was accompanied by an increase in cell-based ALP activity (mean: plus 40% (SD 24); p < 0.01) (Figure 1). Cell density was lower following CTS compared with CON (mean: minus 20% (SD 15), p = 0.02; Figure 1), which was similar to the changes found in cell viability tested by XTT assay (minus 18% (SD 18), p < 0.05). As a consequence, the phenotypic osteogenic response in terms of cell-based ALP activity and cell numbers (μM/min) was not statistically significantly altered by CTS on average (mean: plus 11% (SD 29), p = 0.3), but ranged widely between minus 31% and plus 60% (Figure 1). No significant sex differences were found in any of the variables mentioned previously.

**Table 3.** Genotypic effects of cyclic tensile strain (CTS) on hMSCs. Differential gene expression for cell cycle-specific nuclear antigen Ki67 (MKI67), collagen type I (COL1A1), alkaline phosphatase (ALPL), runt-related transcription factor 2 (RUNX2), osteonectin (SPARC), and osteopontin (SPP1) was determined by real-time RT-PCR in unstrained (CON) and strained (CTS) hMSCs.

	Log_2_ – Ratio (CTS/CTR)			
	Median	95 % CI	p-value	X-fold
MKI67	-0.74	–2.03–1.15	0.51	0.4
COL1A1	1.80	1.15–2.25	0.01	3.5
ALPL	1.27	0.28–1.85	0.02	2.4
RUNX2	0.94	0.25–1.53	0.02	1.9
SPARC	2.04	1.30–2.32	0.004	4.1
SPP1	1.48	1.12–2.00	0.005	2.8

### Donor-specific mechano-response of hMSCs

While no statistically significant correlations were found with any of the determined parameters in strained or unstrained as referred to in Ta ble 4 hMSCs (raw data), there was a correlation between the phenotypic ALP response (i.e. per cent relative change between CTS and CON) and the donor's anthropomorphometric characteristics BMI (r = –0.91, p < 0.001) and BW (r = –0.78, p = 0.006) (Table 4A). This mechano-response was also found to be related to fasting levels of leptin (r = –0.68, p = 0.04) and estradiol (r = 0.67, p = 0.05), while all other indices of bone (i.e. osteocalcin, CrossLaps, insulin-like growth factor I, parathyroid hormone, and human growth hormone) and fat metabolism (i.e. triglycerides, cholesterin, glucose, adiponectin, and resistin) did not appear to be significantly correlated to the osteogenic mechano-response (Table 4B). Furthermore, no significant relationship was found at the gene expression level between the physiological characteristics of the donor and the functional characteristics of hMSCs, either in CON and CTS (raw data) or in strain-related changes (data not shown).

**Table 4.** Physiology of donors and the phenotypic mechanoresponse of hMSCs. Possible relationships between donor's physiology, characterized by (A) anthropomorphometric and (B) biochemical variables and the phenotypic characteristics in terms of ALP activity of unstrained hMSCs (CTR, raw data) as well as the response to cyclic tensile strain in terms of relative change of ALP activity were determined by linear correlation analysis.

			Pearson r	
Donor's physiology	Mean value (SEM)		ALP (CTR) (μM/min)	Relative change of ALP (CTS/CTR) **^a^**
Antropomorphometric characteristics				
Age (years)	66 (4.4)	f: 65 (5.6)	0.49 (p=0.2)	–0.56 (p=0.1)
			m: 67 (7.4)	
BW (kg)	74 (4.9)	f: 68 (4.2)	0.49 (p=0.2)	–0.78 (p=0.006)
			m: 81 (6.9)	
BMI (kg/m^2^)	28 (1.9)	f: 28 (2.9)	0.25 (p=0.7)	–0.91 (p<0.001)
			m: 28 (2.6)	
Biochemical characteristics				
Bone metabolism				
Osteocalcin (ng/mL)	10.6 (1.4) **^a^**		0.06 (p=0.9)	–0.13 (p=0.7)
Cross laps (pmol/L)	4049 (955)		–0.01 (p=1)	–0.01 (p=1)
Parathyroid hormone (pg/mL)	27.1 (3.3)		0.43 (p=0.2)	–0.34 (p=0.4)
Insulin-like growth factor-I (ng/mL)	137.4 (30)		0.59 (p=0.1)	0.57 (p=0.2)
Human growth hormone (ng/mL)	1.20 (0.69)		–0.13 (p=0.7)	0.35 (p=0.3)
Fat metabolism				
Triglycerides (mg/dL)	405 (162)		–0.11 (p=0.8)	0.33 (p=0.4)
Cholesterin (mg/dL)	137 (15.7)		–0.12 (p=0.8)	0.33 (p=0.4)
Glucose (mg/dL)	84.2 (9.4)		–0.21 (p=0.6)	–0.43 (p=0.3))
Leptin (ng/mL)	8.3 (2.4)		0.05 (p=0.9)	–0.68 (p=0.04)
Adiponectin (ng/mL)	5729 (779)		0.14 (p=0.7)	–0.51 (p=0.2)
Resistin (ng/mL)	76.0 (14.4)		0.66 (p=0.05)	0.03 (p=0.9)
Estradiol (pg/mL)	13.5 (3.7)		–0.23 (p=0.6)	0.67 (p=0.05)

**^a^** Osteocalcin: female 7.8 (1.8) ng/mL, male 12.9 (1.5) ng/mL; p = 0.04

### Donor's age, obesity, and the mechano-response of hMSCs

**Figure 2.**
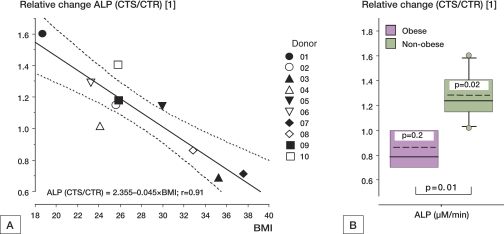
Relationship between the phenotypic mechanoresponse of hMSCs and donor's body mass index (BMI) A. The mechanoresponse in terms of the relative change in ALP activity (μM/min) between unstrained (CTR) and strained (CTS) hMSCs was a function of donor's BMI (r=-0.91, p<0.001). B. This was significantly altered in obese (n=4) compared with non-obese (n=6) patients. Dashed lines show mean (CTR) values.

There was an inverse correlation between the CTS response = relative change (CTS/CTR) in cell-density and donor's age (r = –0.71, p = 0.02), but age did not affect the cell-based ALP activity (r = –0.05, p = 0.9). While there was only a trend of reduced phenotypic osteogenic mechano-response with age (r = –0.56, p = 0.1) (Table 4A), this was less in obese patients than in non-obese patients (p = 0.01) (Figure 2B).

## Discussion

The importance of physical factors in the development and maintenance of bone tissue has been recognized for many years (Harada and Rodan 2003) and it has been convincingly shown that bone cells are sensitive to physical stimuli. Recently, tensile forces of up to 4,300 μstrain were reported to support osteogenic differentiation of hMSCs under appropriate culture conditions (Mirza et al. 2007), which is in accordance with our findings, where cyclic tensile strain of up to 3,000 μstrain was sufficient to initiate an osteogenic stimulus at the gene expression level and at the level of ALP activity (Table 3 and Figure 1). With regard to biological regeneration, cell differentiation and the number of cells are the critical factors for an appropriate response, and—since CTS affected both factors—the phenotypic osteogenic response to CTS may be best reflected by ALP activity at the culture-well level (μM/min). Interestingly, the hMSCs did not respond well to mechanical strain in some of the patients, while this osteogenic response was quite pronounced in others. Most importantly, this variability was found to be highly related to the BMI of the donor (Figure 2).

The pronounced intrinsic heterogeneity of multipotent hMSCs is well known and is thought to represent the lineage hierarchy, where some of the cells are multipotent stem cells while others are more restricted primitive progenitor cells of several cell lineages at various stages of differentiation (Aubin and Triffitt 2002). Thus, it might be speculated that the BMI-related mechano-response we found may be due to a commitment of hMSCs independently of CTS, for example, simply due to the number of fat cells in the culture of obese patients. However, this can be ruled out for several reasons: it is very unlikely that cells at passage 4–6 cells would be committed in a cell-specific differentiation pathway. This has been convincingly demonstrated by large-scale gene expression analyses (Tremain et al. 2001, Kulterer et al. 2007, Scheideler et al. 2008) and is further supported by the fact that we detected no messages for adipo- or chondrocytic markers (i.e. CSPCP and COL2A1) in CON and CTS. Most importantly, due to the study design, any commitment independent of CTS would also have affected the hMSCs in CON; but none of the genotypic and phenotypic parameters in CTS and CON (raw data) were related to the mechano-response as determined by paired analysis, or were significantly correlated to any of the donor's physiological characteristics (Table 4). Consequently, our findings suggest that physiological factors associated with donor's BMI may not necessarily affect the overall osteogenic potential of hMSCs, but will severely hamper the osteogenic response of hMSCs to mechanical strain, which may be of clinical importance.

It is well established that mechanical forces are major determinants of bone mass (Harada and Rodan 2003). Although absolute bone density is known to be higher in obese individuals, it has been demonstrated that when adjusted for body weight, these individuals suffer lower relative bone area and bone mass than non-obese individuals (Goulding et al. 2000), which may also be responsible for a paradoxical increase in fracture risk (Strotmeyer et al. 2005). This is in accordance with previous findings (Weiler et al. 2000), which showed that body fat (relative to weight) negatively influenced bone mineral content and bone density during growth, thus compromising the attainment of peak bone mass. In addition, there is evidence of increased risk of implant failure after lower limb joint surgery in obese patients, particularly after total joint replacement of the knee (Winiarsky et al. 1998, Foran et al. 2004, Berend et al. 2005, Amin et al. 2006).

It is important to consider that all hMSC samples were isolated from bone marrow at the iliac crest. Thus, it seems unlikely that local conditions related to primary diagnosis indicating the need for elective surgery would account directly for the mechano-resistance in our cohort. Indeed, the process of aging as well as obesity itself are accompanied by multiple changes in an individual's whole body physiology, whereas each of them has the potential to severely affect the functional properties of hMSCs within the marrow “stem cell niche” (Caplan et al. 1998). Although interpretation of the data may be limited due to the small sample size, some of the results found from correlation analysis are quite coherent and provide some tentative suggestions regarding this challenging issue. Indeed, adipose tissue is being increasingly recognized as a secretory organ that releases bioactive peptides known to affect a number of physiological functions in bone metabolism also. As expected, fasting leptin levels correlated with BMI, but were also inversely related to the phenotypic ALP response (Table 4), which contrasts with previous findings that leptin is capable of directly stimulating osteoblast differentiation via leptin receptors (Thomas and Martin 2005). Hence, the inverse correlation between the CTS response and leptin levels is likely to reflect its co-linearity with BMI. Based on our findings, the adipokines adiponectin and resistin cannot be attributed to the BMI-linked mechano-response too, but a weak direct correlation was found with the levels of 17β-estradiol. This is an interesting finding because estrogen is known to be a strong determinant of bone mass in both women and men, and has been convincingly shown in vitro and in vivo to critically mediate mechanostimulatory effects via estrogen receptor (ER)-alpha (Lee et al. 2003). However, the correlation with the osteogenic mechano-response was weak; thus, as yet unknown variables related to the donor's fat metabolism are likely to play crucial roles in the impaired responsiveness of hMSCs to mechanical stimuli.

While our study was designed with a view to exclusion of covariate factors beyond mechanical straining, we should mention that weak associations may be easily missed due to the sample size and the intrinsic variability of undifferentiated hMSCs. Although causality cannot be proved by correlation analysis, the finding that the variability in genotypic and phenotypic characteristics of hMSCs was not related to BMI—while this was a strong predictor of the phenotypic osteogenic mechano-response under controlled in vitro conditions—is strongly suggestive of a systemically upstream process of imprinting of hMSCs within the donor's marrow stem cell niche. For this, further investigation is necessary, since obesity-related mechano-resistance of hMSCs may possibly be of clinical significance for disturbances in bone metabolism, bone-healing, and the risk of implant failure.
